# Factors influencing feeding practices of extreme poor infants and young children in families of working mothers in Dhaka slums: A qualitative study

**DOI:** 10.1371/journal.pone.0172119

**Published:** 2017-02-16

**Authors:** Ashraful Kabir, Mathilde Rose Louise Maitrot

**Affiliations:** 1 Dushtha Shasthya Kendra, Dhaka, Bangladesh; 2 Department of Social and Policy Sciences, University of Bath, Bath, United Kingdom; Institut de recherche pour le developpement, FRANCE

## Abstract

**Background:**

Nutritional status differs between infants and young children living in slum and non-slum conditions—infants and young children living in City Corporation slums are likely to have worse nutritional status compared to those from non-slums. Furthermore, families in slums tend to engage female labor in cash-earning activities as a survival strategy; hence, a higher percentage of mothers stay at work. However, little is known about feeding practices for infants and young children in families with working mothers in slums. This study aims to understand the factors that determine feeding practices for infants and young children living in families with working mothers in Dhaka slums.

**Methods:**

This study adopted a qualitative approach. Sixteen In-depth Interviews, five Key Informant Interviews, and Focused Group Discussions were conducted with family members, community leaders, and program staff. Method triangulation and thematic analyses were conducted.

**Results:**

Feeding practices for infants and young children in families with working mothers are broadly determined by mothers’ occupation, basis civic facilities, and limited family buying capacity. Although mothers have good nutritional knowledge, they negotiate between work and feeding their infants and young children. Household composition, access to cooking facilities, and poverty level were also found to be significant determining factors.

**Conclusion:**

The results suggest a trade-off between mothers’ work and childcare. The absence of alternative care support in homes and/or work places along with societal factors outweighs full benefits of project interventions. Improving alternative childcare support could reduce the burden of feeding practice experienced by working mothers and may improve nutritional outcomes.

## Introduction

In recent decades, rapid urbanization with fast population growth has become inescapable in Bangladesh. The demographic dynamics of Bangladesh suggest that the country is undergoing rapid urbanization, particularly due to rural-urban migration [1]. According to the World Bank, Dhaka—the capital city of Bangladesh—has an estimated 300,000–400,000 migrants annually, and is consequently ranked as the fasted growing mega-city in the world [2]. Dhaka has become a popular destination due to the continuous flow of migrants driven by frequent natural disasters, landlessness, farming land shortages, and an agriculture labor surplus. Such rapid urban growth contributes to widespread urban poverty and many poor people live in slums. The population of Dhaka City is likely to approach 21 million by 2015, and one third of that population will be settled in slums [3]. Furthermore, families in slums rarely rely on a single income source due to the high cost of living coupled with irregular income and low wages [4]. Instead, families tend to engage female labor in cash-earning activities as a survival strategy. Approximately 50% of adult women have participated in the labor market to reduce financial pressure in slum households [4, 5].

Under these circumstances, Bangladesh has made substantial progress in recent years. Reduced maternal and child mortality rates, immunization coverage, increased contraceptive use, and greater life expectancy at birth are major successes. Despite these advances, the persistence of malnutrition and nutrition-related health problems remain serious concerns. The number of children under the age of five with severe acute malnutrition (SAM) and moderate acute malnutrition (MAM) is estimated to be 600,000 and 1.8 million, respectively. The prevalence of chronic malnutrition among children under five years old is 41% [6].

However, there are differences in nutritional outcomes between infants and young children (IYC) from families living in slum versus non-slum conditions. For example, the latest urban health survey (UHS) conducted in 2013 shows the prevalence of stunting (height-for-age below -2 SD), underweight, and wasting among IYC are 50%, 43%, and 19%, respectively, in City Corporation slums, and 33%, 26%, and 16%, respectively, in City Corporation non-slums [7]; while, nationally the corresponding figure is 36%, 33% and 14% among under 5 children[8]. According to the Bangladesh Demographic and Health Survey 2014, disparities are also evident in under-five mortality rate (U5MR) and Infant Mortality Rate (IMR)—nationally 46 and 38 per 1000 live births, and 57 and 49, in City Corporation slum families, respectively [7, 8]. Approximately 25% of children in City Corporation slums 6–23 months of age are fed in accordance with IYC feeding (IYCF) practices; in contrast, the corresponding figure for the same age group in City Corporation non-slums is 40%. Furthermore, the report indicates that inadequate and inappropriate feeding is the most obvious factor contributing to poor nutritional status of IYC in slum families.

Thus, the question remains—what factors determine feeding practices for IYC in City Corporation slums? Although existing studies reported nutritional information and statistics in slums, they present little explanation for these results. Some international studies analyzed socio-economic and cultural factors that influence feeding practices within different settings [7;9–13], but these studies do not focus on families of working mothers in slums. The existing literature lacks investigations about what factors influence feeding practices that result in poor nutritional status for IYC from families with working mothers in City Corporation slums. Hence, this study aims to fill the knowledge gap to help policy makers, planners, and program managers improve factors associated with feeding practices for IYC in families with working mothers in City Corporation slums in Dhaka, Bangladesh.

## Methods and materials

### Study population and settings

This study was conducted in two slums (*Korail* and *Kamrangirchar*), in a Dhaka City Corporation area where *Dushtha Shasthya Kendra (DSK)*, a Dhaka based NGO, has been implementing a comprehensive health, nutrition, and livelihood development project. *Korail* slum is surrounded by wealthy neighborhoods (*Gulshan* and *Bonani*) and *Kamrangirchar* slum is located in the area of old Dhaka (*Puron Dhaka*). *Korail* is the largest slum and encompasses approximately 85 acres of land owned by the state-run Bangladesh Telecommunications Company Limited (BTCL). Although there are no official population statistics, some secondary sources show that the slum has approximately 80,000 inhabitants [14]. Poor and inadequate civic facilities, such as irregular water and gas supply, poor road connectivity, and narrow walking space, are common features of slum settings. Overcrowding, a filthy environment, violence, and influence of local *Mastan*, (thug) conflict are prevailing phenomena in the slum. The *Kamrangirchar* slum is built on privately owned land. This is the largest cluster of slums located in the Kamrangirchar peninsula with nearly 400,000 inhabitants. That low-lying area was once used as a dumping ground; therefore, it has been developed as a result of waste dumping in the last few decades. Industrial toxic substances were directed to the river; consequently, inhabitants are living in a severely polluted environment. The inhabitants are poor immigrants from various parts of the county. Overcrowding pushes them to share minimal space. For example, a 100 square-foot room is shared by 5–7 people. Basic civic facilities are poor in the slum.

### Project description

*Dushtha Shasthya Kendra* (DSK) has been implementing a scale-fund project in two urban slums in Dhaka under a project titled “Moving from extreme poverty through the economic empowerment of extreme poor families” since 2009 to support government initiatives to improve livelihood outcomes in extremely poor families. The objective of the project is to lift 30,000 extremely poor households out of poverty. The project incorporated nutritional support in 2012. The intervention included counseling pregnant and breastfeeding mothers to breastfeed exclusively, providing breastfeeding demonstrations, and promoting awareness of complementary feeding from six months onwards, food hygiene, hand washing, sanitary practice, and dietary diversity. Micronutrient powder (MNP) supplements were also provided to children aged 7–23 months. Approximately 56% of female-headed families are self-employed, indicating that a high proportion of women are engaged in cash-generating activities outside the home.

### Data collection procedure

Data were collected between November 2014 and February 2015. The author conducted in-depth interviews (IDI) and focus group discussions (FGD). Sixteen IDI were conducted with the primary family caregiver in the household (e.g., mother, father, relative) to determine their IYC feeding experience and practice. We considered these as infant and child who belong to 0–23 months of age. Six FGD were conducted to explore communal attitudes and perception of factors that affect IYC feeding practice. We also conducted five key informant interviews (KII) with project staff (Nutrition Officer cum Master Trainers; NOMT) who are assisting with intervention implementation to understand various aspects of IYC feeding practices in slums. Participants were purposely chosen, which is a common guiding principle of qualitative research design [15]. The sample size was fixed based on the principle of data saturation—we stopped collecting data when no new information, dimension, or aspect emerged. Table 1 summarizes the methods in detail.

**Table 1:** Methodology and Participants.

Methods	Participants	Field Sites
In-depth Interview (16)	Mothers involved in cash earning activities	*Kamgangirchar*, *Korail*
Key Informant Interview (5)	Community Based Organization (CBO) leaders, Nutrition Officers	*Kamgangirchar*, *Korail*
Focus Group Discussion (6)	Community Pusti Kormi (CPK), Household members (fathers, mothers, and grandmothers)	*Kamgangirchar*, *Korail*

### Data management and analysis

Interviews were conducted in Bangla, the mother tongue of the interviewer and participants, and were recorded electronically. Data were analyzed using a thematic analysis technique. After verbatim transcription, the interviews were translated into English. Broad recurring themes were identified from the interviews. Next, we generated codes based on the meaning statement of the interview, and then looked for clusters of codes. To increase coding validity, independent codes were created from a few interviews by the first author and a research assistant. Finally, triangulation of interviews and focus group discussions was used to validate the data.

### Ethical considerations

This study was reviewed and approved by the ethical review committee of Shiree under the research administration. The research administration of Shiree reviewed human subjects involved in the proposed protocol prior to beginning the study. Participation was voluntary. Balancing risk involvement, practicability, and ethics, we sought verbal consent. In slums, asking for a signature or thumbprint implies that the person is getting involved in a very serious and/or complex issue, which might discourage participation. We developed a consent form containing the objective, importance, and possible pros and cons of the study, which were explained to participants. Considering the circumstances, we proposed to take verbal consent of the participants, which was approved by ethical review committee. The author read the consent form and informed participants of their right to information, and then encouraged them to inquire about the study. Verbal consent was audio-recorded.

## Results

The results of this study are presented in two parts. The first part shows socio-demographic participant characteristics, and the second part shows theme-based factors associated with IYC feeding practices. As shown in Table 2, the mean age of participants (women) was 30.57 (SD±5.15) years. The majority of mothers at both sites had no formal schooling, and mean years of schooling was 1.3 (SD±0.2) in *Kamrangirchar* and 4.5 (SD±1.2) in *Korail*. The combined mean age of IYC is around 14 months (SD±1.6) was relatively similar between the two sites. Nuclear family structures were predominant at both sites (12/16). The combined mean family size was relatively similar across sites, with each family composed of five members, on average.

**Table 2:** Socio-demographic characteristics of the participant (IDI: n = 16).

Variables	Fields Sites		Combined
	*Kamrangirchar(n = 10)*	*Korail(n = 6)*	
**Age of the women in years (mean** ±SD)	28.6±5.6	33.84±4.7	30.57±5.15
**Schooling of women**			
No formal schooling (*n*)	6	1	7
1–5 years (*n*)	4	3	7
6–10 years (*n*)	0	2	2
Schooling in years (mean ±SD)	1.3±0.2	4.5±1.2	2.5±1.3
**No. of adult proxy care givers (%)**	4 (40%)	3 (50%)	7 (43.75%)
**Occupation (*n*)**			
Street plastic vendor	2	0	2
Housemaid	3	2	5
Garment workers	1	2	3
Scavenger	1	0	1
Factory worker (shoe)	2	0	2
Vegetable seller	0	2	2
Others	1	0	1
**Marital Status (***n*)			
Married	9	6	15
Widowed	1	0	1
**No. of years living in slum (mean**±SD)	5.9±2.4	6±2.7	5.94±2.9
**Age of the child in months (mean** ±SD)	14.5±1.4	13.33±1.3	14.06±1.6
**No. of infant and child living per household (mean** ±SD)	2.9±1.2	2±1.3	2.82±1.6
**Husband schooling years**			
No formal schooling (*n*)	4	2	6
1–5 years (*n*)	5	3	8
6–10 years (*n*)	1	1	2
Schooling in years (mean ±SD)	2.6±1.2	2.8±1.2	2.6±1.4
**Family type**			
Nuclear (*n*)	8	4	12
Extended (*n*)	2	2	4
**Family size (mean** ±SD)	5.1±1.2	5±1.3	5.06±1.5
**Religion**			
Muslim (*n*)	10	5	15
Hindu (*n*)	0	1	1

Table 3 shows some characteristics of the FGD participants. Six FGDs were conducted: one among 6 *Community Pusti Kormi* (CPK) in *Kamrangirchar* (mean age, 29, SD±7 years), one among 7 CPK in *Korail* (mean age, 32, SD±9 years), one among 7 mothers and grandmothers in *Korail* (mean age, 25, SD±6 years), one in *Kamrangirchar* among 8 mothers and grandmothers (mean age, 24, SD±8 years), one in *Kamrangirchar* among 8 fathers (mean age, 35, SD±7 years) and, one in *Korail* among 6 fathers (mean age, 27, SD±10 years)

**Table 3:** Socio-demographic backgrounds of participants in focus group discussions.

Focus group discussion	Age of the participants in years (mean ±SD)	Location	Number of respondents	Type of participants
I	29±7	***Kamrangirchar***	6	Community Pusti Kormi (CPK)
II	32±9	***Korail***	7	Community Pusti Kormi (CPK)
III	25±6	***Korail***	7	Female household members (mothers and grandmothers)
IV	24±8	***Kamrangirchar***	8	Female household members (mothers and grandmothers)
V	35±7	***Kamrangirchar***	8	Male household members (fathers)
VI	27±10	***Korail***	6	Male household members (fathers)

Three main themes emerged from the data, and are presented below.

### Mother’s livelihood and IYC feeding patterns

Mothers, CBO leaders, and program personnel including CPK *(Community Pushti Kormi)* and Nutrition Officers commonly reported that IYC in slum families were more likely to consume processed food due to their mothers’ work. Mothers reported that they were engaged in a variety of cash-earning activities that made cooking at home difficult. Consequently, IYC were commonly fed cheap processed food to meet food requirements. Such cheap food items are processed in small and medium food factories, contain poor nutritional density, and are often prepared in unhygienic conditions. Table 4 shows three categories of processed foods that are cheap, available, and easy to prepare and serve.

**Table 4:** Consumption of processed food by IYC in the last 24 hours of interview.

Categories of Foods	Common items	Sources	Explanations for feeding choice
Liquids	Fruit juice, *Akher Sharbat* (a drink made with sugar cane, lemon leaf, and ice),	Street vendors, street corner/grocery shops.	Available, cheap, convenient, perceived as healthy
Semisolid	*Chutney*, *Sandesh*, *Mawa*, *Malai* (sweetmeats made locally with sugar, water, flavor) Curd, Ice-cream, lollipop, and pickles	Street vendors, street corner/grocery shops.	Available, cheap, convenient, the infants seemed to like eating them
Solid	Chips, biscuits, *roti/chapatti* (flat breads), *Chitaipitha* (rice cakes), *bhapapitha* (steamed rice cake), alur chap *jhalmury* (Spicy puffed rice), puri, *singara*, *rutiporata*, *chotpoti*, *bhelpuri and*, *phuchka* (popular snacks made with potatoes, vegetables, boiled eggs and traditional spices, and tamarind sauce), *biryani* (rice with meat), damaged/leftover fruits. traditional cakes, bread,	Street vendors, street corner grocery shops.	Available, cheap, easy to prepare and feed

Although young mothers are generally aware of the nutritional value of different kinds of food, appropriate feeding times, and hygiene practices, they are often inconsistent about feeding IYC accordingly because they are away from home due to their work. They reported learning about these topics through NGO interventions like information sharing, counseling, and demonstration. In an IDI, one mother said:

“Apa (CPK) taught me how to feed a child and suggested exclusive breastfeeding until the child is six months of age. She showed me how to administer home-cooked food to the child after six months using a bati (small bowl). She also gave me a pusti packet (nutrition supplement) that is mixed with rice to increase its nutritional value. I try to follow her instructions.”

However, while mothers are working away from home, IYC are mostly looked after by older siblings (4–10 years old). It is common for mothers to leave a little cash with the caregiver so they can buy food from local groceries or vendors to feed themselves and the IYC. There are also some home-cooked meals at home, but caregivers (elder sibling, neighbor, elderly relative/kin) are generally “unable” to properly feed IYC because they lack knowledge about nutrition and hygiene needs. Apart from older siblings, grandmothers (women’s mothers-in-law) mostly take care of IYC while mothers are at work. Mothers-in-law are perceived to have minimal nutritional knowledge and are often likely to neglect IYC. In many cases, IYC are not adequately fed in a timely manner by these caregivers. Fathers rarely cooked for IYC. In Bengali culture, men do not generally participate in food preparation at home because this is perceived as a woman’s duty. Although women living in slums work and earn, men do not prepare food or care for their IYC while mothers are at work. Only one father reported that he once administered liquids to his child with the help of an older sibling. Generally, fathers preferred to buy food from local groceries or vendors, or fed the child whatever home-cooked meal was left before the mother went to work. This care pattern was corroborated by numerous respondents. For example, one mother said the following in an IDI:

“My infant was in good health until I started my business as a hawker. Since then, his health has been deteriorating. He is getting smaller, losing weight, and getting ill very often. His older sibling is not capable of looking after him properly because she is also a child. Sometimes, I leave a little money for them to buy, biscuits, bread, chips, etc. I do not have any other option because I have to be away for long hours due to work.”

Similarly, a CPK from FGD explained:

“In many cases, the responsibility of taking care of IYC, which includes feeding, cooking, and bathing, is transferred to an older sibling aged 6–7 years. This is where the main problem lies, because the caregiver does not have any knowledge of hygiene or nutrition. The young child herself needs care, how can she take care of another baby? The mothers often return home late after a day of strenuous labor and have little energy left to breastfeed the child.”

Similarly, one nutrition officer stated the following in a KII:

“How can we expect that such a little child [7–10 years old] to properly feed the infant and young child? Many times he himself eats non-family food. How can he protect the young child from the introduction of cheap, unhygienic, and poor nutritional food?”

Usually, the mothers earn very little through their works as they are mostly involved in low-paying informal job. Among them, the mothers working at garment earn comparatively bigger that ranged from US$ 40 to US$65 a month. The rest of the mothers such as housemaid or scavengers reported to earn less than US$ 30 a month. Majority of participants reported that the mothers had little and/or no scope for spending their wage to buy quality food as their husbands spend it. Usually the husbands spend it to pay house rent, basic utilities, food grains, cloth, transport, and health care. One of the CPKs in KII stated

“The women enjoy little and/or no freedom in spending their income to buy quality food for infant and young child because, they are struggling to survive. They earn very little that limit their desire to spend beyond basic need. Besides, they have to give this money to their husbands as the husbands are the key decision makers and care taker of the families.”

### Breastfeeding and mothers’ livelihoods

Participants reported that mothers often skip breastfeeding due to working time constraints. This results in infants consuming semi-solid and even solid food before reaching six months of age. Nearly two thirds (9/16) of mothers interviewed told us that they were unable to breastfeed their IYC adequately and in a timely manner because their babies are sleeping or not hungry when they leave for work early in the morning. In an IDI, one mother stated:

“How can I regularly breastfeed my child? I need to go to work so that my family can survive. I had to start working when my child was only four months old. I could not breastfeed and had to choose other options.”

However, some mothers informed us that they extracted and stored milk before leaving their homes, as program individuals (mostly CPK) suggested and demonstrated. The purpose of extracting is to save milk for later use. However, caregivers did not adequately feed that milk to IYC in a timely manner. Participants commonly reported that they (caregivers) hardly maintained timeliness and frequency of feeding. Half (3/5) of the CPK reported similar things. In an FGD, one CPK said:

“We advise the mothers to extract breast milk before leaving for work. However, many mothers are unable to do so as they leave for work in the morning. We have noticed that infants often become dependent on cheap processed food such as suji and milk before reaching six months of age.”

In a KII, another nutrition officer said:

“Even when mothers stored breast milk at home before leaving for work, the caregiver could not administer it properly. Often, the milk was not properly stored, or the caregiver could not understand how many times or when to feed the child or what milk temperature was ideal for feeding. This happened, of course, because they were IYC themselves, and lacked adequate knowledge on the matter.”

There are some exceptions. In an IDI, one mother reported that her child was breastfed beyond six months of age by minimizing working hours:

“I continued exclusively breast feeding until my child was six months of age. Supplementary food/milk was out of the question because I could not afford it. Because I work as street vendor, I tried to work hours that would give me the minimum income I needed and came back home early to breastfeed my child.”

Most respondents reported that they hardly managed to maintain the minimum number of meals required for their young IYC (twice for breastfed infants 6–8 months, three times for breastfed IYC 9–23 months, and four times for non-breastfed IYC 6–23 months) [16]. Additionally, multiple caregivers, including older siblings, mothers, grandmothers, and fathers, took care of the IYC at different times during the day and fed the child according to their own convenience, not according to the needs of the child. It is not common for relatives to be taken temporarily from their village home for the purpose caring for IYC while mothers are at work. However, it is common for the relative to go back to their home, so the family must bring another relative. In a KII, one nutrition officer stated:

“The child is sometimes taken care of by an older sibling, sometimes by the husband, and sometimes by relatives. The relatives are taken from their village homes to provide care when mothers are absent. But, relatives may go back to their village at any time.”

Consequently, young infants are often fed cheap, processed food from local groceries, and feeding times are inadequately spaced. Although some participants stated that the IYC were normally fed a variety of food based on availability and cost, the minimum dietary diversity intake requirement (foods that contain protein, carbohydrates, fat, vitamins, and minerals) seemed to be inadequate in most families interviewed. IYC were less likely to be fed milk products and animal foods. Limited purchasing power and lack of knowledge of the proxy care givers are main reasons for nutrition deficiencies. One of participants in a KII informed

“Although the mothers have gained good level of knowledge as we get them involved in program intervention, the proxy caregivers often remain beyond. Therefore, the proxy caregivers lack level of knowledge about the proper feeding while the mothers are at works. … Poor level of knowledge about feeding might result in nutritional deficiency such as underweight and/or stunting among infant and young child”

Food items from local stores are introduced to the IYC along with home-cooked meals, but this still fails to maximize dietary variety due to poor nutritional quality. In *Kamrangirchar*, some major internal chicken organs like gizzards, kidneys, hearts, brains, and livers are cheap. Street vendors collect and sell these organs on roadsides while restaurants and chain shops reject them. However, such chicken items are not available in *Korail*. Thus, chicken organs are a popular food item in slum families in *Kamragirchar*. In an IDI, one mother said:

“In slums, you can buy leftover poultry organs at a low price, even cheaper than some vegetables. Many people sell it on street corners.”

### Basic civic facilities and feeding patterns

Around two thirds (10/16) of participants in IDIs and majority in KIIs and FGDs reported that a crucial reason for not attaining minimum nutritional requirements was the lack of facilities in slums. We found that although most families had gas connectivity, an inadequate supply forced them to skip cooking. In both slums, gas burners are shared. Consequently, it is difficult to cook multiple times a day. This problem is exacerbated by an inadequate gas supply. Usually, gas pressure is adequate late at night to the morning; thus, most families try to cook at this time. Mothers often wait in line to get a turn. As a result, women often cook large quantities of food, and it is common for women who leave for work in the early morning to miss the chance to use the shared burner. Apart from that, the family has limited/no use of firewood for cooking because it is expensive. Families seem to use firewood for cooking when it is the only option. Otherwise, they avoid using firewood for cooking because it is expensive. Instead, they resort to buying food like cheap bread and butter from local vendors, which is also fed to the IYC. Such observations came from multiple participants (both mothers and CPK). In an IDI, a nutrition officer said:

“Meals are cooked once a day due to inadequate gas supply in the slums. And those that use firewood to cook tend to avoid cooking from time to time because of the high cost of firewood. This leads to buying processed food items for themselves and their IYC.”

In an IDI, one of the mothers in *Kamrangirchar* said:

“If you consider the cost of firewood, it is nearly the same as the cost of the food that will be cooked. I bought 40 kg of firewood, so I will be calculating about when I will use it. I have the ability to buy another 40 kg this week. I cannot use that firewood randomly. Rather, I consider using it when I really need to cook. It may be wise to use it at most once a day.”

In an IDI, another mother in *Korail* explained how the cost of firewood leads to feeding processed food:

“Five kg of firewood costs 50 taka. I cannot afford it every day. I can only buy three kg, and that is not sufficient for cooking more than once a day. So, I buy chewing gum, chocolate, and lollypops to feed my child. These products are very cheap and you can feed three or four young babies with only 10 taka.”

Moreover, after long hours of physical work, mothers are exhausted and do not have enough energy to care for and feed their babies. Because they have other household duties to perform, childcare is in competition with cooking, washing clothes, dusting, cleaning, and socializing. Some participants struggle to provide care for their infants because there is no one else available to look after them at home, and there is no childcare support (especially for young mothers with their first baby). This reduces the likelihood of maintaining proper feeding. Furthermore, babies are exposed to unfavorable environments that cause sickness. One nutrition officer explained the situation in a KII:

“I found a mother who was engaged in heavy construction work at night. She had to take her one-month-old baby with her to work because there was nobody at home to take care of the child. The newborn thus got a cold and fever.”

Because good quality processed food is unaffordable, mothers are pushed to choose food items for young babies that lack proper nutritional properties. More than half of the participants reported opting for cheap food items for their babies because they are cheap, available, and suitable for feeding IYC. They make this decision even though they know that inadequate feeding practices coupled with frequent introduction of cheap processed food at home might deteriorate nutritional intake in young babies.

## Discussion

This paper discussed some factors that influence feeding practices for IYC living in families with working mothers in slums. Our data show that mothers in slums are aware of the nutritional requirements of their IYC and appropriate feeding practices. Yet, their engagement in market labor when they do not have adequate childcare support at home or work negatively affects nutritional intake. Household composition, access to cooking facilities, and level of poverty were also were found to be significant factors.

The information gathered in this study suggests that mothers living in slums have significant knowledge about nutrition, breastfeeding, childcare, and early initiation to complementary food. Mothers were informed through community-based interventions (notably the *DSK-shiree* project), routine counseling visits to young mothers, demonstrations, and follow-ups. This is consistent with another study. For example, Afsana et al. [17] found that nearly 68% of mothers had appropriate knowledge of micronutrients powder (MNP) in Bangladesh because of community-based message delivery (through counseling by community health workers). Our study suggests that despite having nutrition-related information, regular micronutrient powder supplements, and breastfeeding opportunities, mothers were not consistently breastfeeding or giving complementary food in a timely manner due to their work outside the home.

Income generating occupations were identified as the main barriers to appropriate child feeding practices when no alternative childcare support was available either at home or work, and this combined with other idiosyncratic factors creates an unfavorable situation for IYC. Family composition is one exacerbating factor. The predominant nuclear family structure in slums reduces the scope of alternative support and possible caregivers. The dependency on people outside the family is high (e.g., relatives or neighbors). Families are also largely characterized by low income [18] (in the latest survey the mean per capita household income was 78 Bangladeshi taka); they often have small networks and rely on multiple income sources and precarious jobs (night work, construction work, long working hours), which jeopardize their efforts to provide adequate food to their young babies. In many cases, mothers leave their babies for short time periods with proxy caregivers—mostly older siblings, and occasionally neighbors or relatives from another village—who may not be consistent in timely feeding or who may place little or no emphasis on nutritional considerations [9]. Young babies may not be fed properly in terms of breastfeeding or complementary food intake because regular caregivers are absent. Some families could mobilize support by bringing a relative from another village for short periods of time.

Our findings are consistent with global studies that found that mothers’ employment without adequate alternative care (from a family member or at the work place) negatively impacted nutrition status in IYC [19–24]. For example, a study at Surabaya, Indonesia [21] found significantly lower height-for-age Z-scores (HAZ) for IYC with working versus non-working mothers. Weight-for-age z-scores (WAZ) and HAZ were also significantly lower for IYC with mothers who worked versus non-working mothers. A study in Mali [25] found that young children aged 12–36 months had lower HAZ if their mothers were involved in income generating activities. However, maternal involvement in cash crop production was found to be positively associated with WAZ scores in IYC, and there was a negative correlation with energy intake from non-breast milk foods [25]. Nakahara et al. [19] investigated the association between childcare support availability and nutrition outcomes of young babies aged 10–24 months in a peri-urban setting in Nepal. The lack of adult childcare support was linked to an increased risk of malnutrition in children from working and non-working mothers; however, the risk of malnutrition was higher in young children with working mothers. Yeleswarapu et al. [26] investigated the nutritional status of young children with employed and unemployed mothers in urban slums in Andhra Pradesh, India. They found that weights and heights were significantly higher in children younger than five years of age from families with employed versus unemployed mothers. Mothers are unlikely to enjoy the full benefits of interventions due to the absence of alternative childcare support at home and/or work, long working hours, limited capacity and opportunities to use civic facilities, and negligence or lack of nutritional knowledge in proxy caregivers. Similarly, a qualitative study by Nair et al. in rural settings in the Dangarpur district of Rajasthan, India [22] indicated that mothers’ employment required compromises in infant care and feeding, such as breastfeeding and timely feeding. Furthermore, infants cared for by a family member (e.g. mother-in-law) had a higher chance of being neglected and having impaired nutritional outcome. Mittal et al. [27] found that mothers’ occupation type also influenced nutritional status of IYC in slums in India. In a cross-sectional study conducted in slums, they found that IYC were more likely to be underweight and stunted when mothers were involved in formal work versus not involved in formal work (46.15% versus 37.8% and 58.97% versus 44.8%, respectively). Mothers’ engagement in cash earning activities resulted in less time spent on food preparation and breastfeeding, consistent with a study by Moser et al. in Germany [20]. However, some studies found that mothers’ participation in income generating activities had positive impacts on nutritional outcomes in IYC. For instance, Lamontagne et al. [28] showed that weight and height were higher in children aged 12–18 months with employed versus unemployed mothers in low urban communities in Nicaragua. However, the study further showed that proxy caregivers in working mothers’ families engaged in hand washing practices while feeding the IYC. Rathnayake et al. [29] found that in Sri Lanka, socio-economic factors, especially mothers’ income, had significant positive effects on total caloric intake (CI). Time allocation for childcare such as breastfeeding, food preparation, and feeding contributed to nutritional status of IYC. A study in a low income peri-urban settings in Kenya [30] found the young babies aged 0–2 years were more likely to be malnourished (stunted) if they were from families with working mothers.

However, there is some evidence to suggest that maternal employment may not be the most important factor for malnutrition. Joanne [31] reviewed and analyzed 50 published papers on the relationship between women’s work and nutritional status of young child in developing countries and concluded that there was little evidence that maternal employment had a negative impact on child nutrition. He concluded that the impact of women’s engagement in cash-generating activities was complex and not systematic, and that it could have both positive and negative effects on young children’s nutrition (e.g., growth of the families’ purchasing power increases capacity to buy more/better food). Another study by Popkin [32] in 34 rural barrios in Laguna, Philippines, showed that young babies living in families where mothers were engaged in market activities were consuming more calories and protein than the bare minimum. These results might differ from our findings due to caregiver characteristics, because the predominant caregivers were older siblings who might provide more effective care than younger siblings. In Dhaka slums, the number of working women has increased with no provision for adequate care facilities at home or work while they are working. Such conditions lead to negative IYC feeding practices, which are further exacerbated by socioeconomic slum characteristics. Therefore, efforts should be made to support adequate feeding of IYC while mothers are outside the home.

## Limitations of the study

Results of this study are based on small sample size. The mothers also had good level of knowledge about IYC feeding due to the nutritional program intervention in these slums. The mothers in other slums are likely to have poor level of knowledge about the feeding practice of IYC. Therefore, the results might not be transferable in other settings. Nonetheless, considering the point of data saturation, we believe that study provides an in-depth understanding the factors affecting feeding practice of extreme poor infants and young children in families of working mothers in Dhaka slums.

## Conclusion

The objective of this study was to understand the factors that influence IYC feeding practices in families with working mothers where adequate alternative childcare support is absent. This study showed that there is a trade-off between mothers’ work and infant and child feeding. Mothers’ works coupled with other societal and economic factors prevent mothers from taking full advantage of program interventions when alternative support is not available. Experimental studies on home-based daycare centers within minimal resource allocation, promotion of non-financial supports (e.g., links with other NGO that operate daycare centers, promote awareness and facilitate community mobilization activities), and community-based working/livelihood options are needed to elucidate ways to improve the situation.

## Supporting information

S1 File Guideline for In-depth Interview (IDI). (DOCX)

S2 File Guideline for Focused group Discussion (FGD). (DOCX)

S3 File Guideline for Key Informant Interview (KII). (DOCX)
