# Supplementary material for: Factors influencing feeding practices of extreme poor infants and young children in families of working mothers in Dhaka slums: A qualitative study
Source: PLoS One. 2017 Feb 16;12(2):e0172119. doi: 10.1371/journal.pone.0172119 (PMC5312963; doi:10.1371/journal.pone.0172119)
Supplement: S1 File — (DOCX) [file pone.0172119.s001.docx]

Guideline for In-depth Interview (IDI)

1. Socio-demographic information of the participants (name, age, occupation, education, religion, family structure, income, number and age of infant and child in the household)
2. Would you please say something about infant and child nutrition? (What is meant by nutrition? What affect nutrition? How can it be improved? Who can contribute in improving nutrition status etc.?)
3. How is your infant and child health? (Describe elaborately health condition of the infant and child, disease frequency etc.)
4. Please say something about the feeding of your infant and child while you are work place. What type food they are fed? How and when they are fed? Who feed them (Please discuss elaborately when, how, why and why not?)
5. In your opinion, how your works effect the feeding of your infant and child? (Why and why not?)
6. In your opinion, what are the important factors that affect feeding of your infant and child (Basic utilities, buying capacity/income, firewood, family composition etc.)? Why do you consider those factors as important and why not?
7. How do these factors affect the feeding of your infant and child? (Why and why not?)
8. In your opinion, how these conditions can be improved? (Why and why not?)
