# Supplementary material for: Factors influencing feeding practices of extreme poor infants and young children in families of working mothers in Dhaka slums: A qualitative study
Source: PLoS One. 2017 Feb 16;12(2):e0172119. doi: 10.1371/journal.pone.0172119 (PMC5312963; doi:10.1371/journal.pone.0172119)
Supplement: S2 File — (DOCX) [file pone.0172119.s002.docx]

Guideline for Focused group Discussion (FGD)

1. Socio-demographic information of the participants (name, age, occupation, education, religion, designation, residing slum)
2. How is infant and child health living in slum households?
3. How is infant and child nutrition living in slum households?
4. How the infant and child in slum households are fed while the mothers are at work places? What type food they are fed? How and when they are fed? Who feed them (Please discuss elaborately when, how, why and why not?)
5. What is the effect of mothers’ works on infant and child feeding? (Why and why not?)
6. What are other important factors that affect infant and child feeding (Basic utilities, buying capacity/income, firewood, family composition etc.)? Why it is important and why not?
7. In your opinion, what can be done to improve these conditions? (Why and why not?)
