# Supplementary material for: Factors influencing feeding practices of extreme poor infants and young children in families of working mothers in Dhaka slums: A qualitative study
Source: PLoS One. 2017 Feb 16;12(2):e0172119. doi: 10.1371/journal.pone.0172119 (PMC5312963; doi:10.1371/journal.pone.0172119)
Supplement: S3 File — (DOCX) [file pone.0172119.s003.docx]

Guideline for Key Informant Interview (KII)

1. Socio-demographic information of the participants (name, age, occupation, education, religion, designation)
2. What is your opinion about infant and child health living in slum households?
3. What is your opinion about infant and child nutrition living in slum households?
4. How the infant and child living in slum households are fed while the mothers are at work places? What type food they are fed? How and when they are fed? Who feed them (Please discuss elaborately when, how, why and why not?)
5. How the mothers’ works effect the feeding of infant and child? (Why and why not?)
6. What are important factors that affect feeding of your infant and child (Basic utilities, buying capacity/income, firewood, family composition etc.)? Why these are important and why not?
7. How do these factors affect the feeding of infant and child? (Why and why not?)
8. In your opinion, what can help improve these conditions? (Why and why not?)
